# Therapeutic drug monitoring (TDM) of atazanavir in pregnancy

**DOI:** 10.1186/1758-2652-13-S4-P177

**Published:** 2010-11-08

**Authors:** LJ Else, V Jackson, M Brennan, J Breiden, C Weldridge, S Coulter-Smith, DJ Back, SH Khoo, J Lambert

**Affiliations:** 1 University of Liverpool, Department of Pharmacology, Liverpool, UK; 2 The Rotunda Hospital, Dublin, Ireland; 3 Mater Misericordiae University Hospital, The Catherine McAuley Research Centre, Dublin, Ireland; 4 University College Dublin, Dublin, Ireland

## Background

Pregnant women experience physiological changes during pregnancy resulting in clinically significant alterations in antiretroviral pharmacokinetics (PK). Therefore, achieving and maintaining optimal plasma concentrations of antiretroviral drugs is essential for maternal health and minimising the risk of mother-to-child transmission of HIV.

## Purpose of the study

To describe atazanavir/ritonavir (ATV/r) PK during pregnancy.

## Methods

In this prospective, open labelled study, pregnant HIV-positive women received ATV/r as part of their routine pre-natal care. Demographic and clinical data were collected, and ATV plasma concentrations [ATV] were determined in the first (T1) and/or second (T2) and/or third (T3) trimester using HPLC-MS/MS (LLQ = 0.05 µg/mL). Postpartum (PP) sampling was performed where applicable. Antepartum (AP) and PP PK parameters were compared using a One-way ANOVA (for independent data sets) and a paired t-test (for paired data).

## Summary of results

From January 2007 31 women (25 black African) were enrolled in the study. All received ATV/r at standard dose of 1 tablet once daily (300/100 mg od). 10/31 women initiated ATV/r treatment during pregnancy. Median (range) gestation at initiation in these patients was 24 weeks (7-35). Median (range) baseline CD4 count was 393 cells/µL (153-869) and 17 patients had a baseline plasma viral load of < 50 copies/mL. [ATV] were determined in 10/31 (T1); 17/31 (T2); 27/31 (T3) and 21/31 (PP) patients. Time of TDM sampling and [ATV] (geometric mean; 95%CI) are given in the Table. 2/17 patients at T2, 2/27 (T3) and 2/21 at PP had concentrations <LLQ (suggesting non-adherence). [ATV] were lower AP relative to that observed at PP (Table 1). Equally, in a paired analysis of 17 patients (T3 vs. PP), [ATV] were significantly reduced at T3 (P=0.0005).

**Table 1:** 

	T1 (n=10)	T2 (n=17)	T3 (n=27)	PP (n=21)	P value
[ATV], µg/mL	1.03 (0.49-2.53)	0.75 (0.59-1.45)	0.66 (0.63-1.03)	1.17 (1.09-1.81)	0.06

CV, %	108	84	59	55	-

Time of sampling, h	14.6 (12.5-17.5)	18.0 (16-21.8)	19.0 (17.6-21.9)	19.1 (17.4-22.3)	0.102

## Conclusions

Although ATV concentrations were reduced in the third trimester, standard ATV/r dosing did achieve therapeutic levels (>150 ng/mL) both antepartum and postpartum, suggesting the current regimen is appropriate in pregnancy.

